# Multidisciplinary team approach for CKD-associated osteoporosis

**DOI:** 10.1093/ndt/gfae197

**Published:** 2024-09-24

**Authors:** Ditte Hansen, Hanne Skou Jørgensen, Thomas Levin Andersen, Ana Carina Ferreira, Aníbal Ferreira, Renate de Jongh, Satu Keronen, Heikki Kröger, Marie Hélène Lafage-Proust, Leena Martola, Kenneth E S Poole, Xiaoyu Tong, Pieter Evenepoel, Mathias Haarhaus

**Affiliations:** Department of Nephrology, Copenhagen University Hospital – Herlev, Copenhagen, Denmark; Institute of Clinical Medicine, University of Copenhagen, Copenhagen, Denmark; Institute of Clinical Medicine, Aarhus University, Aarhus, Denmark; Department of Nephrology, Aalborg University Hospital, Aalborg, Denmark; Molecular Bone Histology (MBH) lab, Department of Clinical Research, Faculty of Health Sciences, University of Southern Denmark, Odense, Denmark; Department of Pathology, Odense University Hospital, Odense, Denmark; Danish Spatial Imaging Consortium (DanSIC), University of Southern Denmark, Odense, Denmark; MBH lab, Department of Forensic Medicine, Aarhus University, Aarhus, Denmark; Department of Nephrology, ULS São José Lisbon, Portugal; Universidade Nova de Lisboa- NOVA Medical School-Nephology, Lisbon, Portugal; Department of Nephrology, ULS São José Lisbon, Portugal; Universidade Nova de Lisboa- NOVA Medical School-Nephology, Lisbon, Portugal; Department of Endocrinology and Metabolism, Amsterdam UMC location Vrije Universiteit Amsterdam, Amsterdam, The Netherlands; Amsterdam Gastroenterology Endocrinology Metabolism Research Institute, Endocrinology, Metabolism and Nutrition, Amsterdam, The Netherlands; Abdominal Center, Department of Nephrology, Helsinki University Hospital and University of Helsinki, Helsinki, Finland; Kuopio Musculoskeletal Research Unit (KMRU), University of Eastern Finland, Kuopio, Finland; Department of Orthopedics, Traumatology, and Hand Surgery, Kuopio University Hospital, Kuopio, Finland; INSERM U1059, CHU, Université de Lyon, Saint-Etienne, Lyon, France; Abdominal Center, Department of Nephrology, Helsinki University Hospital and University of Helsinki, Helsinki, Finland; NIHR Cambridge Biomedical Research Centre & Department of Medicine, University of Cambridge, Addenbrooke's Hospital, Cambridge, UK; Kuopio Musculoskeletal Research Unit (KMRU), University of Eastern Finland, Kuopio, Finland; Department of Microbiology, Immunology and Transplantation; Nephrology and Renal Transplantation Research Group, KU Leuven, Leuven, Belgium; Department of Medicine, Division of Nephrology, University Hospitals Leuven, Belgium; Division of Renal Medicine, Department of Clinical Science, Intervention and Technology, Karolinska Institutet, Karolinska Universitetssjukhuset, Stockholm, Sweden; Diaverum AB, Malmö, Sweden

**Keywords:** bone biopsy, chronic kidney disease–mineral and bone disorder, multidisciplinary team, osteoporosis, renal osteodystrophy

## Abstract

Chronic kidney disease–mineral and bone disorder (CKD-MBD) contributes substantially to the burden of cardiovascular disease and fractures in patients with CKD. An increasing arsenal of diagnostic tools, including bone turnover markers and bone imaging, is available to support clinicians in the management of CKD-associated osteoporosis. Although not mandatory, a bone biopsy remains useful in the diagnostic workup of complex cases. In this special report, the European Renal Osteodystrophy (EUROD) initiative introduces the concept of a kidney–bone multidisciplinary team (MDT) for the diagnosis and clinical management of challenging cases of CKD-associated osteoporosis. In 2021, the EUROD initiative launched virtual clinical-pathological case conferences to discuss challenging cases of patients with CKD-associated osteoporosis, in whom a bone biopsy was useful in the diagnostic workup. Out of these, we selected four representative cases and asked a kidney–bone MDT consisting of a nephrologist, an endocrinologist and a rheumatologist to provide comments on the diagnostic and therapeutic choices. These cases covered a broad spectrum of CKD-associated osteoporosis, including bone fracture in CKD G5D, post-transplant bone disease, disturbed bone mineralization, severely suppressed bone turnover and severe hyperparathyroidism. Comments from the MDT were, in most cases, complementary to each other and additive to the presented approach in the cases. The MDT approach may thus set the stage for improved diagnostics and tailored therapies in the field of CKD-associated osteoporosis. We demonstrate the clinical utility of a kidney-bone MDT for the management of patients with CKD-MBD and recommend their establishment at local, national, and international levels.

## Graphical Abstract

**Graphical Abstract:**
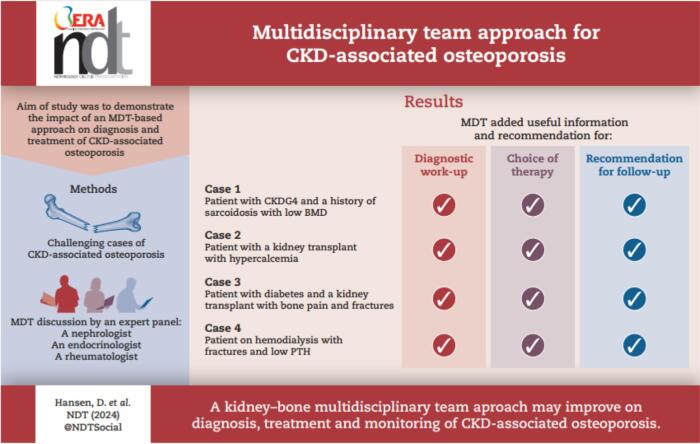

## INTRODUCTION

Chronic kidney disease—mineral and bone disorder (CKD-MBD) contributes substantially to the increased morbidity and mortality in CKD [1–3]. Patients with CKD have an exceptionally high risk of fractures, which increases progressively as kidney function declines [4–6]. Despite recent advances in diagnosis and therapy, fractures remain an important cause of hospitalizations and mortality in advanced CKD.

Decisions regarding diagnosis and treatment of CKD-associated osteoporosis remain challenging due to the frequent convergence of CKD-MBD with primary and secondary osteoporosis, each conferring specific bone traits [4, 7, 8]. Thus, kidney–bone multidisciplinary teams (MDTs), integrating expertise from different clinical specialties, are evolving in an increasing number of centers as a novel approach to management of complicated cases. These MDTs can involve nephrologists, endocrinologists, rheumatologists, pathologists, radiologists and endocrine surgeons, among others. Access to histomorphometric analysis of bone biopsies is an advantage, but not a necessity.

Renal osteodystrophy (ROD) is the collective term for the broad spectrum of changes in bone metabolism attributed to CKD. Changes in bone turnover, mineralization and volume are the prominent features that can be identified by histomorphometric analysis of a bone biopsy, which remains the gold standard for a diagnosis of ROD. The current diagnostic system reports bone turnover (T) as high, normal or low, mineralization (M) as normal or abnormal, and volume (V) as high, normal or low [9]. As bone biopsies are invasive, and both the bone histomorphometric analysis and the clinical interpretation require special expertise, they are seldomly performed in everyday clinical practice [10]. However, in a subset of patients, bone biopsies are helpful, as the exact histomorphometric diagnosis will influence the choice of therapy or may reveal unexpected bone pathology.

The European Renal Osteodystrophy (EUROD) Initiative, a European network of clinicians and researchers with a particular interest in CKD-associated osteoporosis, was started in 2016 by the European Renal Association CKD-MBD Working Group. The intention of the EUROD Initiative is to improve the clinical management of ROD, and to promote the bone biopsy as a clinically useful diagnostic tool in specific cases. To increase the awareness of how kidney–bone MDTs can improve the diagnosis and treatment of CKD-associated osteoporosis, the EUROD initiative organizes virtual clinical-pathological case conferences as a multi-disciplinary online forum to discuss challenging cases of CKD-associated osteoporosis. A selection of cases from these conferences have been gathered in the present special report to demonstrate the impact of an MDT-based approach on diagnosis and treatment of CKD-associated osteoporosis.

## METHODS

Four cases from three different European countries (Denmark, Finland, France) presented at the virtual EUROD clinical-pathological case conferences [11] were selected to cover the broad spectrum of CKD-associated osteoporosis. The managing clinicians were invited to provide, in a structured manner, the following pieces of information: (i) a short description of the clinical problem, medical history, relevant laboratory and imaging data, and indication for bone biopsy, (ii) results of the bone histomorphometry, including the TMV classification, and (iii) treatment and follow-up.

The four cases were distributed to an international MDT-like panel (one nephrologist: P.E., one rheumatologist: K.E.S.P., and one endocrinologist: R.J.). These were asked to provide comments or recommendations based on the following three questions. (i) Would you have found other examinations useful in the given situation? (ii) Would you suggest any other treatment in the given situation? (iii) Would you highlight any learning points or areas for future research based on this case? The expert panel then discussed their comments in a virtual meeting and agreed on joint take home messages for each case.

### Case 1: a patient with CKD G4 and a history of sarcoidosis presenting with low BMD and high bone-specific alkaline phosphatase

A 43-year-old white male had CKD G4 caused by sarcoidosis with pulmonary, hepatic and renal involvement since he was a teenager. He was underweight (49 kg; body massindex 18.4 kg/m^2^), with no nutritional deficiencies. Due to long-term prednisolone treatment (currently 15 mg/day combined with azathioprine) a dual-energy X-ray absorptiometry (DXA) scan was performed, showing very low BMD with T-score of –4.8 at the lumbar spine and –3.2 at the femoral neck. He had no prior fragility fractures, and there were no vertebral fractures on a spinal X-ray. Kidney function was stable for the last 4 years with an estimated glomerular filtration rate (eGFR) of 25 mL/min/1.73 m^2^. Current biochemistry revealed hyperparathyroidism [intact parathyroid hormone (PTH) 32 pmol/L; reference range (RR) 1.3–6.9], normal calcium (total calcium 2.48 mmol/L) with several previous episodes of hypercalcemia, and low-normal phosphate (0.99 mmol/L). Bone alkaline phosphatase (BALP) was elevated at 42 µg/L (RR 5.5–22.9 µg/L) and had been stable for several years (35–51 µg/L). Calcitriol levels were high (110 pg/mL; RR 20–80), 25-hydroxyvitamin D (25OHD) was previously low (13 nmol/L), but had increased to 97 nmol/L under medical therapy with cholecalciferol 50 000 IU every 3 months. The patient was on no other CKD-MBD-specific treatment. The indication for a bone biopsy was very low BMD with suspicion of high bone turnover and/or a mineralization defect.

**Figure 1:**
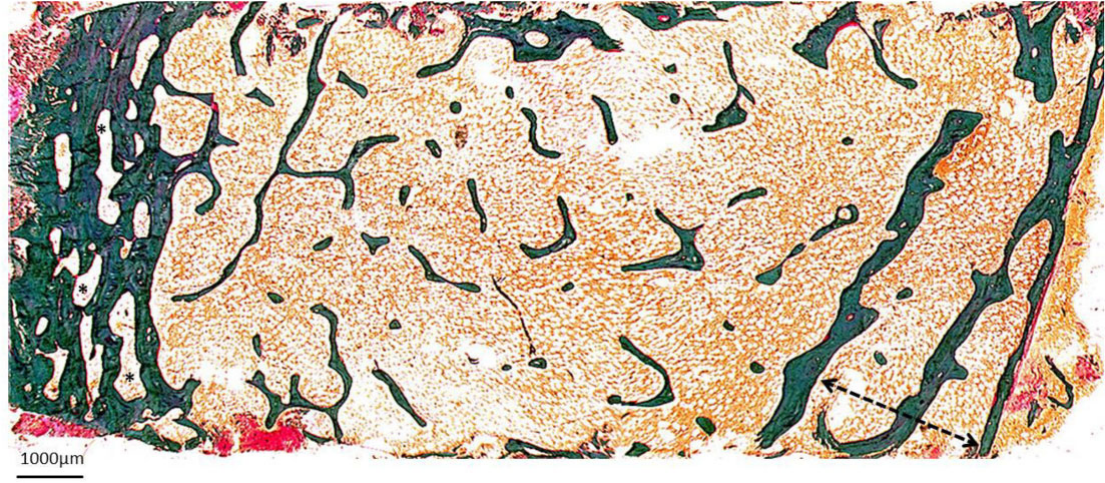
Case 1: bone biopsy from a patient with CKD G4 and a history of sarcoidosis presenting with low BMD and high bone-specific alkaline phosphatase. The biopsy revealed low trabecular bone volume with increased cortical porosity. Bone turnover was normal to high, with increased bone resorption. There was no mineralization defect. Full section of iliac crest sample. Goldner-stained section (5×). Asterisks illustrate the increase in cortical porosity of the outer cortex. Dotted line marks the space where the inner cortex was before being trabecularized and resorbed. Short arrows illustrate the extreme thinning of the remaining inner cortex.

Bone histomorphometry showed very low trabecular bone volume with increased cortical porosity. Bone turnover was normal to high, with increased bone resorption. There was no mineralization defect. Resorption parameters (eroded surface and osteoclast surface) were elevated confirmed by TRAP staining. The pathological diagnosis was trabecular and cortical osteoporosis (Fig. 1). TMV classification: turnover: normal-high; mineralization: normal; volume: low.

After the bone biopsy, the patient was treated with high-dose oral vitamin D supplementation (cholecalciferol 50 000 IU every 2 months) as well as anti-resorptive therapy with risedronate 35 mg/week to protect against further bone loss. His prednisolone was not discontinued due to active sarcoidosis (calcitriol serum levels remained high). One-year follow-up: intact PTH did not increase under combination therapy of cholecalciferol and risedronate while BALP showed a 30% decrease after 6 months of treatment and remained at this level. The kidney function remained stable with eGFR at 22 mL/min/1.73 m^2^.

#### Comments from the experts, Case 1 (Table 1)

**Table 1:** Expert panel take home messages, Case 1.

• Collect information on long-term bone history; peak bone mass determines adult BMD
• Acknowledge other fracture risk factors besides low BMD, for example malnutrition and low body weight
• For BALP as a bone turnover marker, potential confounding factors include effects of liver dysfunction, chronic inflammation, and the possibility of a bone mineralization defect
• Before starting denosumab in patients with CKD, it is important to discuss that therapy cannot be discontinued without the risk of rebound bone loss and increased fracture risk. This can be prevented by off-label use of a bisphosphonate, when denosumab is withheld
• Patients with sarcoidosis can be supplemented with calcium and native vitamin D if the sarcoidosis is adequately treated, and calcium level is normal

Endocrinologist: in this case, sarcoidosis was diagnosed and (high-dose) prednisone treatment started when the patient was quite young. Thus, the sarcoidosis itself, the corticosteroid treatment, and effects of lifestyle such as less physical activity, may have negatively affected the peak bone quality and mass. It is important to take these factors, as well as the current weight and height, into account when interpreting the DXA measurement. Taking into account kidney function and the very low BMD, denosumab may have been considered as first choice treatment. The patient has a very low BMD and the positive effect of denosumab on BMD persists during long-term treatment, i.e. for up to 10 years [12], in contrast to oral bisphosphonates, where the additional effect of treatment after 5 years on BMD seems small [13]. With oral bisphosphonates the chance of reaching a BMD out of the osteoporotic range are low, whereas with denosumab the treatment may be continued until BMD scores are acceptable. Importantly, discontinuation of denosumab therapy should always be followed by bisphosphonates to prevent rebound bone resorption. Initiating with an anabolic treatment such as romosozumab before anti-resorptive treatment will increase BMD more than vice versa in non-CKD populations. However, the lack of data on romosozumab in the CKD population limits its use. Attention should also be given to current lifestyle, including physical activity (mechanical loading is essential to increase BMD) and a healthy diet with a sufficient calcium intake (i.e. approximately 1000 mg per day). Bone turnover markers may be useful for monitoring short-term treatment response.

Rheumatologist: in sarcoidosis, *c*holecalciferol is well tolerated by patients and is regularly used once hypercalcemia has been excluded. Vitamin D deficiency should not be permitted to develop, albeit with the caveat that urine calcium should be measured to exclude hypercalciuria after commencing cholecalciferol therapy [14]. This case exemplifies the utility of the bone biopsy. Before biopsy, osteomalacia was a differential diagnosis (elevation in BALP), but bone tissue examination did not show any mineralization defect. The bone biopsy results are given as normal to high bone turnover with increased bone resorption. Hence, the biopsy was informative because the team could administer an anti-resorptive agent as the most appropriate treatment. Follow-up DXA is suggested to monitor the treatment response and plan for a 5-year course of oral therapy.

Nephrologist: the severity of sarcoidosis may, independently of the cumulative dose of glucocorticoid, be associated with bone fragility [15]. Calcium levels should be monitored as markers of activity of the underlying sarcoidosis and glucocorticoids doses should be titrated accordingly. In this case, the high BALP argues in favor of a high bone turnover state. However, both impaired bone mineralization and liver dysfunction with cross-reactivity with the liver isoforms of alkaline phosphatase [16] should be considered as potential confounders. Thus, it would have been valuable to include other non-kidney excreted biomarkers of bone turnover, if available, or perform a bone biopsy as in the present case. A caveat for the use of bisphosphonates in patients with CKD stages G4–5 is the possible risk for worsening kidney function described in observational studies, but evidence from controlled trials is lacking [17, 18].

### Case 2: a patient with a kidney transplant presenting with hypercalcemic hyperparathyroidism

**Figure 2:**
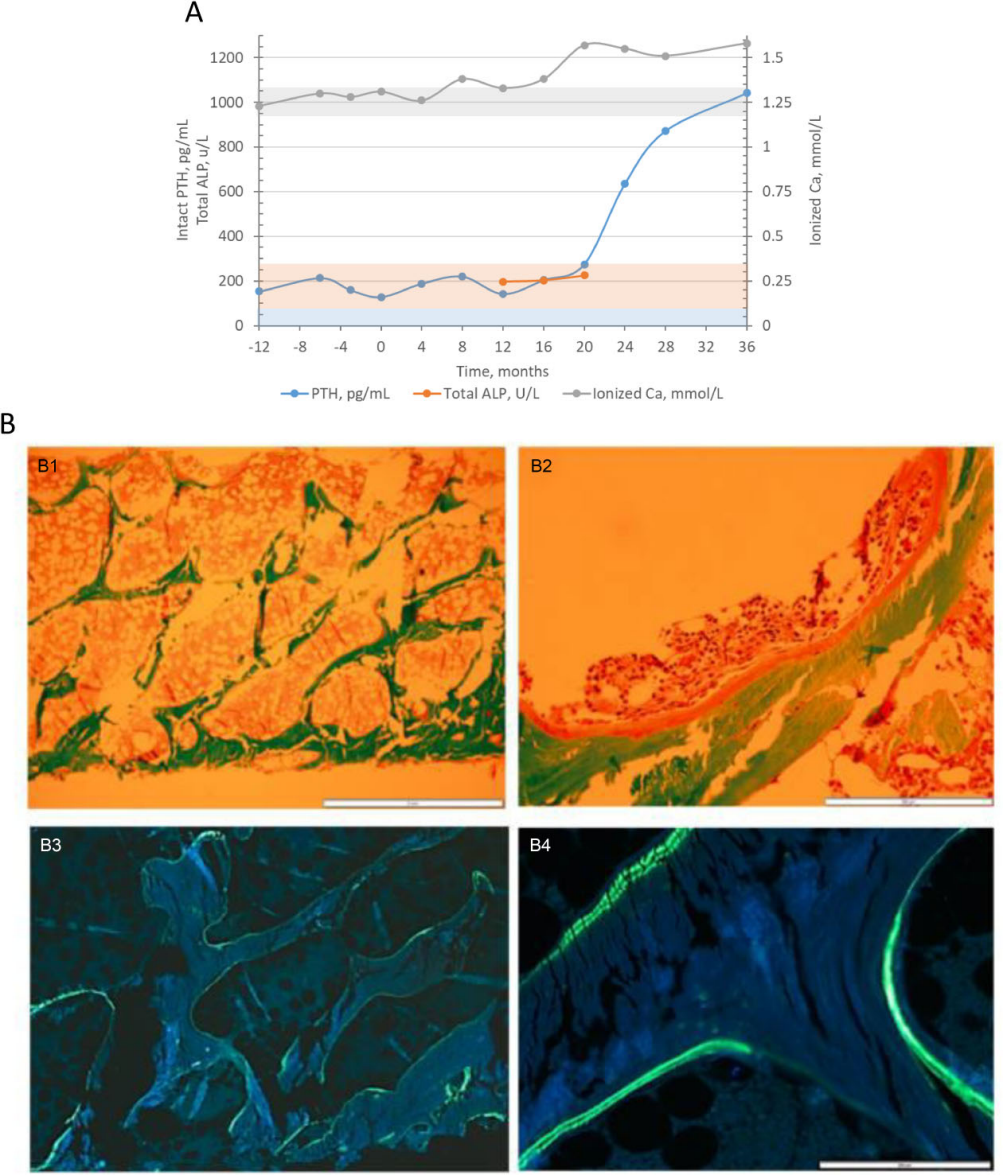
Trends of mineral metabolism parameters and bone biopsy—Case 2: a patient with a kidney transplant presenting with hypercalcemic hyperparathyroidism. (**A**) Trends of mineral metabolism parameters. Ca: calcium. (**B**) A bone biopsy was performed at times 0 and at 24 months. The bone biopsy at 24 months revealed high turnover bone disease. Active bone resorption was increased, while osteoid parameters were in the normal range. UV microscopy showed abundant double labels. TMV classification: turnover: high; mineralization: normal; volume: low. (B1, B2) Masson Goldner's trichrome stain; (B3, B4) fluorescent labelling. Scale bars: 2 mm (B1, B3); 200 µm (B2, B4).

A 34-year-old white male who received a kidney transplant from a living donor 11 years ago and who had stable kidney graft function (CKD G2T), presented with hyperparathyroidism and hypercalcemia. His original kidney disease was chronic pyelonephritis and he had no history of fractures. As part of maintenance immunosuppressive therapy, he received long-term cyclosporine and glucocorticosteroid treatment (cumulative dose of 21 g methylprednisolone). A DXA scan was performed showing low-normal BMD, with a Z-score of –0.5 and T-score of –1.2 at the femoral neck. Biochemistry revealed intact PTH of 13.6–16.8 pmol/L (RR 0.8–7.7 pmol/L) with calcium around the upper normal range (ionized calcium 1.28 to 1.31 mmol/L; RR 1.16–1.30). Alkaline phosphatase was within normal range and no other bone turnover markers or measurements of vitamin D status were available (Fig. 2A). The patient had mild metabolic acidosis (HCO_3_ 21 mmol/L; RR 24–28 mmol/L). The patient did not receive any medical therapy for hyperparathyroidism (calcium or vitamin D supplements, active vitamin D, calcimimetics) or any bone-specific therapies (antiresorptive or anabolic treatment). The indication for a bone biopsy was persistent hyperparathyroidism with hypercalcemia, with suspicion of high bone turnover.

Bone histomophometry showed bone turnover and osteoid parameters within the reference range, low eroded surfaces, and normal double labeling in UV microscopy. The bone volume was low. TMV classification was: turnover: normal; mineralization: normal; volume: low.

After the bone biopsy there was no change in medical therapy; the patient did not receive any specific bone-targeted medications, or any calcium or vitamin D supplementation. After 2 years, severe hypercalcemia with ionized calcium 1.57 mmol/L developed. Intact PTH was moderately elevated at 29.2 pmol/L, with total alkaline phosphatase (ALP) in the upper normal range at 225 U/L (RR 60–275 U/L) (Fig. 2A). Kidney function had only slightly decreased with a current creatinine clearance of 53 mL/min/1.73 m^2^. The indication for a second bone biopsy was the progressive hypercalcemia with only a slight increase of PTH.

Repeat bone histomorphometry indicated high turnover bone disease. In particular, active bone resorption was increased, while osteoid parameters were in the normal range. UV microscopy showed abundant double labels (Fig. 2B). TMV classification: turnover: high; mineralization: normal; volume: low.

After the second bone biopsy, PTH increased rapidly to >100 pmol/L within 6 months, while ionized calcium remained at levels >1.5 mmol/L. A subtotal parathyroidectomy with removal of 3.5 glands was performed 6 months after the biopsy with one gland described as adenomatous (7 g) and the remaining as hyperplasic. A DXA scan 2 years after parathyroidectomy revealed bone gain with an increase in femoral neck Z-score from –0.5 to +0.2. Calcium levels normalized and PTH levels decreased to 18.9 pmol/L.

#### Comments from the experts, Case 2 (Table 2)

**Table 2:** Expert panel take home messages, Case 2.

• A bone biopsy is not mandatory prior to parathyroidectomy either before or after kidney transplantation. Rapidly increasing PTH levels, particularly if unresponsive to medical therapy, should raise the suspicion of autonomy of one or more parathyroid glands
• Trends of biomarkers (including bone turnover markers) are much more informative than single time point measurements and quickly respond to therapeutical interventions
• A low or declining DXA BMD may point to ongoing bone resorption and PTH levels should be assessed as a potential cause
• Fracture risk assessment should include imaging of the vertebrae to reveal any prevalent vertebral fractures
• Be aware of the risk of hungry bone syndrome post parathyroidectomy. Risk assessment should include measurement of total or bone-specific ALP prior to surgery

Nephrologist: persistent hyperparathyroidism with (often mild) hypercalcemia is common after kidney transplantation, but the clinical consequences of this biochemical presentation remains uncertain. The urinary calcium/creatinine clearance ratio may help to distinguish between a primary hyperparathyroidism-like phenotype versus a familial hypocalciuric hypercalcemia (FHH) phenotype, the latter being a prevalent finding in kidney transplant recipients [5]. This distinction is important as it may guide therapy; a low urinary calcium/creatinine clearance ratio (i.e. fractional urinary calcium excretion) indicates hypercalcemia due to renal reabsorption (FHH phenotype), rather than by excessive bone resorption. In this case, the evolution of the mineral metabolism parameters later on, with substantial increases in PTH and severe hypercalcemia, rendered the diagnosis of autonomous hyperparathyroidism so likely that the second bone biopsy could be considered redundant.

Endocrinologist: longitudinal assessment of PTH and calcium is crucial to discriminate ongoing secondary or tertiary hyperparathyroidism versus development of an adenoma, which could be considered a form of *de novo* primary hyperparathyroidism in this patient population. Parathyroidectomy seems a more attractive option than calcimimetics due to the young age of the patient and the steep increase in PTH concentrations in the last year before parathyroidectomy. Measurement of total ALP before parathyroidectomy is warranted as part of the risk estimation of the hungry bone syndrome; other risk factors are increasing levels of PTH and the mass of the excised glands [19–21].

Rheumatologist: in this case, a forearm DXA would be useful to assess cortical bone which is preferentially affected in hyperparathyroidism. Imaging of the parathyroid glands at an earlier stage might have identified an adenoma. Parathyroidectomy was the right choice of treatment. Serial (second) biopsies are unusual in our clinical practice. Here, the second biopsy is indicated by the development of new, clinically important hyperparathyroidism, although it could be argued that it did not influence the parathyroidectomy decision. If there is adequate kidney function, then C-telopeptides of type I collagen (CTX) measurements are a valid way to assess bone turnover. A raised CTX would have been useful at the point of the first biopsy, as indicative of a more systemic bone resorption.

### Case 3: a patient with diabetes and a kidney transplant presenting with bone pain and multiple low-energy fractures

**Figure 3:**
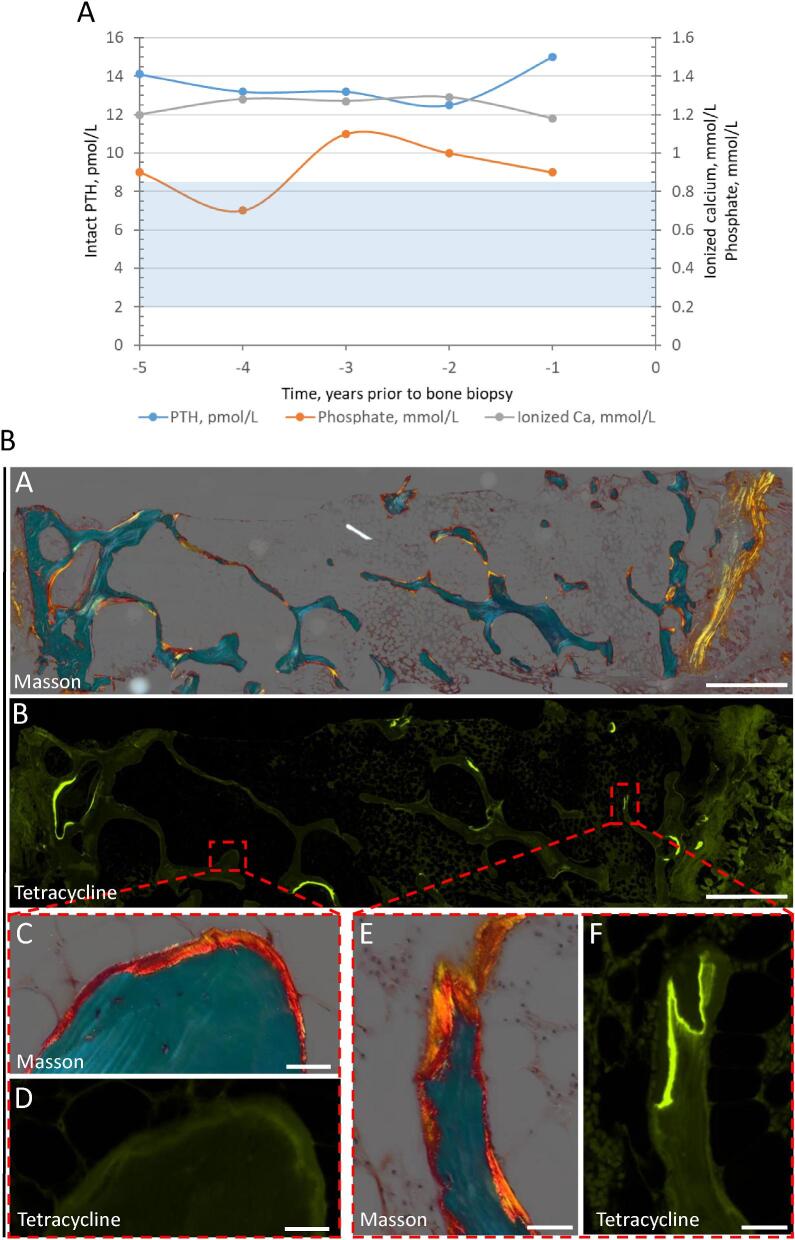
Trends of mineral metabolism parameters and bone biopsy—Case 3: a patient with diabetes and kidney transplantation presenting with bone pain and multiple low-energy fracturs. (**A**) Trends of mineral metabolism parameters. Ca: calcium. (**B**) Bone biopsy showed low trabecular bone volume and thin cortical bone (A) with abnormal mineralization (B, C, E) and low turnover (B, D, F). (A, C, E) Partly polarized light digital microscopy of Masson trichrome staining, showing osteoid surfaces mainly directly deposited on eroded surfaces colonized by a low density of osteoblasts. (B, D, F) Fluorescence digital microscopy of tetracyclin-labeled bone surfaces, which only represented a small fraction of the osteoid surfaces. Scale bars: 1 mm (A, B); 50 µm (C–F).

A 58-year-old white male, with type 1 diabetic nephropathy, who received a kidney transplant 15 years ago and had stable kidney graft function (CKD G3T) presented with bone pain and repeated fractures. At the time of referral, he had experienced spontaneous fractures of the distal tibia 10 years ago, the calcaneus 4 years ago and the second distal tibia 3 years ago. During the last 5–6 years he suffered from severe pain in both feet. A DXA-scan revealed low BMD with a T-score of –2.4 and a Z-score of –0.8 at the lumbar spine and a T-score of –3.2 and a Z-score of –1.2 at the femoral neck. Biochemistry showed slightly elevated intact PTH at 15 pmol/L (RR 1.6–6.9 pmol/L), low-normal ionized calcium at 1.18 mmol/L (RR 1.15–1.30), low-normal phosphate at 0.80 mmol/L (RR 0.76–1.41 mmol/L) and sufficient 25OHD at 86 nmol/L (RR >50 nmol/L) (Fig. 3A). Bone turnover markers were elevated with BALP 55.1 µg/L (RR 8.3–29.4 µg/L), intact trimeric P1NP 149 µg/L (RR 22–114 µg/L) and fasting CTX 727 ng/L (RR 125–1477 ng/L). The indication for a bone biopsy was multiple fractures, bone pain and elevated bone turnover markers, with suspicion of a mineralization defect or high bone turnover.

Bone histomorphometry demonstrated a low trabecular bone volume and markedly thin cortical bone (Fig. 3B). The trabecular bone surfaces were covered in thick osteoid, demonstrating reduced mineralization. Most of the osteoid appeared upon the eroded surfaces, indicating a failure to initiate mineralization. The excessive osteoid surface meant a lower surface for bone turnover and remodeling to occur. TMV classification: turnover: low; mineralization: abnormal; volume: low.

After the biopsy, treatment with alfacalcidol 0.5 µg/day was initiated to improve mineralization. After a few weeks, the pain in both feet disappeared. Re-evaluation by DXA and bone turnover markers is still pending.

#### Comments from the experts, Case 3 (Table 3)

**Table 3:** Expert panel take home messages, Case 3.

• The threshold to perform a bone biopsy should be low if a mineralization defect is suspected, as bone histology is necessary to diagnose this condition
• A mineralization defect should be suspected in cases of bone pain, non-healing or multiple fractures, malabsorption, hypophosphatemia, low 25OHD, acidosis, low PTH, rapidly declining BMD or looser zones on plain X-ray
• A sufficient calcium intake should be ensured for all CKD patients but particularly those with suspected osteomalacia
• The optimal 25OHD level in patients with CKD remains a matter of ongoing debate, with some suggesting to aim for higher levels than recommended in the general population (normal range 50–125 nmol/L)
• Active vitamin D supplements may be considered in osteomalacia, but attention should be paid to avoiding hypercalcemia
• Monitoring the increase in DXA BMD and decrease in BALP may be useful to evaluate the therapeutic response in patients with a proven or suspected mineralization defect. Changes should be rapid (months) following initiation of treatment

Nephrologist: mineralization defect, or osteomalacia, is a dreaded presentation of renal bone disease. Potentially contributing factors include vitamin D deficiency, low serum phosphate or low serum calcium. The risks of a negative calcium balance may not receive sufficient attention, due to concerns of calcium as a driver of vascular calcification. Calcium intake should be estimated and tailored towards a total elemental calcium intake between 800 and 1200 mg/day [22], and potential malnutrition should be addressed. Deficits should be corrected through dietary changes rather than with supplements. In the absence of phosphate retention, dairy products may be the preferred food item as they allow to increase the phosphate exposure simultaneously.

Rheumatologist: clinically apparent osteomalacia in patients with CKD should be confirmed histologically, and treatment is likely to transform the clinical symptoms. A repeat biopsy is usually not needed. Isotope technetium-99m bone scan can reveal further fractures with this degree of osteomalacia, particularly in ribs. The bone biopsy was crucial to understanding what was happening in the bone in this case and provided information that was not available from imaging or bone turnover markers. In this case, 25OHD was in an acceptable range, but treatment with alfacalcidol improved the patient's pain—demonstrating the importance of reduced 1-alpha hydroxylase activity in patients with CKD. Alfacalcidol was the correct choice of therapy.

Endocrinologist: in this case, an assessment of vertebral fractures with imaging would be useful for staging severity and during follow-up. When monitoring treatment response, a DXA may be repeated within 1 year because restoration of the mineralization of bone induces a rapid increase in BMD (within months) in contrast to the slower increase with treatment of osteoporosis (over years). Decrease in bone turnover markers also occurs rapidly (within weeks) after start of treatment.

### Case 4: a patient on hemodialysis presenting with multiple low-energy fractures and low-normal PTH

A 67-year-old white female with two prior kidney transplantations, currently receiving hemodialysis for the past 5 years, was examined due to several low-energy fractures (left ankle fracture, distal forearm fracture, left tibial fracture and upper humeral fracture). She had hypoparathyroidism since a thyroidectomy 15 years ago. A DXA scan demonstrated osteopenia at the lumbar spine T-score –1.8 and Z-score –0.4, and low BMD at the total hip with a T-score of –3.2 and a Z-score –2.1. No X-ray of the vertebral spine was available. Biochemistry was notable for intact PTH levels at 3.0 pmol/L (RR 1.6–6.9 pmol/L), with low-normal ionized calcium at 1.15 mmol/L, high-normal phosphate at 1.3 mmol/L (RR 0.76–1.41 mmol/L), and 25OHD at 53 nmol/L (RR >50 nmol/L). No bone turnover markers were available. No acidosis was present (totalCO_2_ 25 mmol/L; RR 23–31 mmol/L) Medical therapy included a single dose of denosumab, which had been administered 5 years prior to the biopsy, but was withheld after the first dose due to severe hypocalcemia. The indication for a bone biopsy was multiple fractures and assessment of bone turnover before initiating bone-targeted therapy.

**Figure 4:**
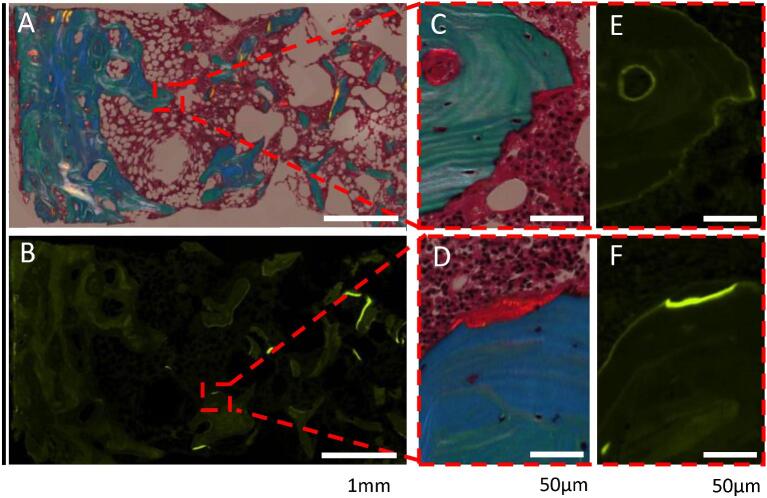
Bone biopsy—Case 4: a patient on hemodialysis presenting with multiple low-energy fractures and low-normal PTH. Bone biopsy showed low trabecular bone volume (A) with normal turnover (B, E, F) and normal mineralization (C, D). Double labels occurred, but this was not included in the high magnification illustration. (A, C, D) Partly polarized light digital microscopy of Masson trichrome staining. (B, E, F) Fluorescence digital microscopy of tetracyclin-labeled bone surfaces. Scale bars: 1 mm (A, B); 50 µm (C–F).

Bone histomorphometry demonstrated a low trabecular bone volume and normal appearing cortical bone (Fig. 4). The trabecular bone surfaces had normal bone turnover with no sign of a mineralization defect. TMV classification: turnover: normal; mineralization: normal; volume: low.

After the biopsy, it was decided to initiate bisphosphonate therapy, although teriparatide was also considered due to the relatively low intact PTH. Re-evaluation by DXA and bone turnover markers is still pending.

#### Comments from the experts, Case 4 (Table 4)

**Table 4:** Expert panel take-home messages, Case 4.

• Non-renally cleared bone turnover markers perform acceptably for the exclusion of low and high bone turnover
• Vertebral fractures may explain a discrepancy between hip and lumbar spine BMD and should always be investigated if there is an indication for DXA
• The threshold to perform a bone biopsy in patients with multiple and/or unexplained fractures should be low. However, not having access to bone biopsies does not justify withholding therapy
• An MDT approach and shared decision-making regarding therapy with anabolic and antiresorptive agents should be considered, taking into account both pharmacokinetics and the individual risk profile

Nephrologist: the use of bone-targeting therapies in CKD is mainly based on expert opinion, as published evidence is very sparse. A bone biopsy may be performed as the state of bone turnover and mineralization can guide the choice of therapy. As a non-invasive alternative, bone turnover markers show high negative predictive value for both high and low bone turnover, and cut-off values to rule out either condition have been suggested for trimeric P1NP, BALP and TRAP5B^23^. Bone turnover markers may also be useful for monitoring the treatment effect. Nonetheless, in this case, a bone biopsy was justified to definitely exclude a mineralization defect, given the history of multiple fractures and a relatively low 25OHD level. Hypocalcemia is a common complication after denosumab therapy, especially if bone turnover is increased, indicating a hungry bone response with rapid skeletal uptake of calcium as bone re-mineralizes. Treatment with calcium and vitamin D, starting 1–2 weeks before the denosumab injection and continuing until calcium normalizes, can decrease the risk of severe hypocalcemia.

Rheumatologist: as to the choice of anti-resorptive therapy in CKD, ibandronate could also be considered (UK Green book recommendation in CKD) [23]. Ibandronate is cleared by both hemodialysis and haemodiafiltration [24], and it can be given as a 3 mg bolus intravenously in the early part of dialysis every 3 months as per the licensed dose. This will give a similar actual clearance of ibandronate as patients with normal kidney function. The safety and efficacy of this treatment for osteoporosis in dialyzed patients is an area for future research; however, in anuric patients the potential risk of worsening kidney function is of no concern. When deciding on “normal turnover” (TMV) it would be helpful to know the eroded surface here.

Endocrinologist: anabolic treatment (teriparatide or romosozumab) could also be considered in this case. The benefit on BMD is highest if the patient starts with anabolic treatment, which should always be followed by antiresorptives to consolidate treatment effects [25]. Romosozumab has a black box warning on the potential increased risk of cardiovascular disease but seems otherwise attractive in this particular patient with fractures of the extremities, and low BMD of the hip, but not the spine. While, teriparatide reduces the risk of vertebral fractures in particular [26], romosozumab also has a positive effect on hip fracture risk. However, data in CKD populations are scarce. Sufficient calcium intake and vitamin D status is extremely important before starting any anabolic or antiresorptive treatment, particularly in CKD patients. Imaging of the spine is warranted to exclude prevalent vertebral fractures as an explanation of the relative high BMD of the spine.

## DISCUSSION

The management of CKD-MBD poses a challenge for clinicians, as multiple organ systems are involved and underlying pathophysiological processes are only partially understood. While current guidelines offer some guidance, the complexity of many cases requires the collaboration of several specialties. In management of high fracture risk in the general population, MDTs in the form of fracture liaison services have been established for some time and have considerably improved outcomes [27, 28]. Here, we present the concept of kidney–bone MDTs for management of challenging cases of CKD-associated osteoporosis. The cases have been retrospectively evaluated by experts after the management by the treating physician and illustrate how an MDT could have added value to the therapeutic choice. Although hypothetical, involving an MDT initially might have changed management of these cases. For example, in Cases 1 and 4, alternative treatment options might have been suggested, in Case 2 the second bone biopsy might have been deemed unnecessary and in Case 3 further diagnostic workup might have influenced management.

Osteoporosis is common in advanced CKD, but is underdiagnosed and undertreated due to the complexity of the underlying bone disease, the lack of evidence from large clinical trials, and fear of interference with existing CKD-MBD, e.g. the aggravation of low bone turnover or a negative effect on residual kidney function [29–31]. The absence of guidance regarding osteoporosis treatment in the 2017 KDIGO CKD-MBD guideline update contributes to the uncertainty regarding fracture prevention in CKD [32]. However, novel evidence has prompted clinically oriented review articles and a consensus statement on management of fracture risk in CKD, offering support for informed clinical decision making [30, 33, 34].

**Figure 5:**
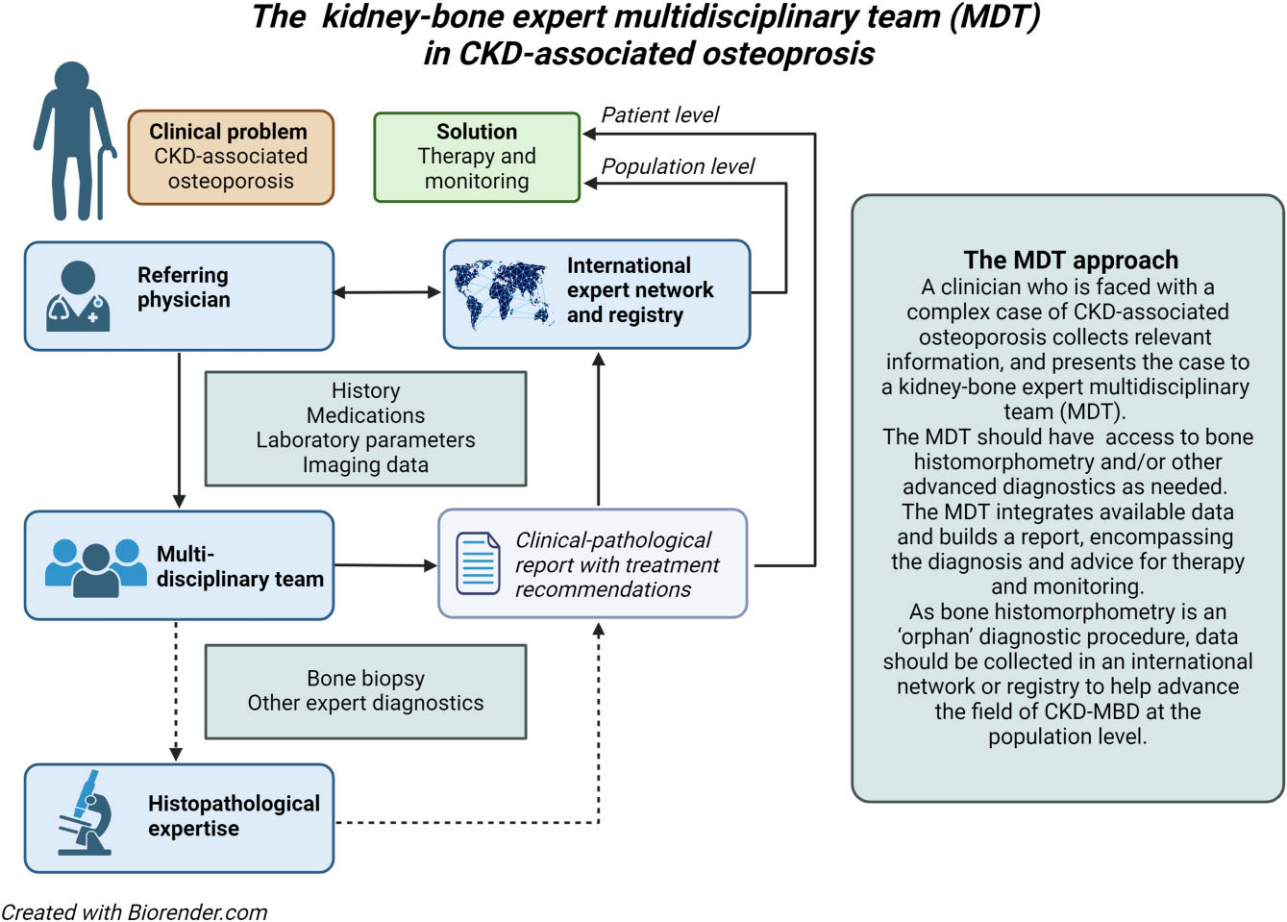
Work flow kidney–bone MDTs.

Decisions on how to regulate the mineral metabolism and whether to institute treatment may often be multifaceted and require a multidisciplinary approach [35]. To facilitate a more active management of fracture risk in CKD, we advocate for the establishment of MDTs in tertiary hospitals, consisting of nephrologists with expertise in CKD-MBD and osteoporosis specialists, and optionally pathologists, radiologists and other healthcare professionals with expertise in CKD-MBD or osteoporosis. These MDTs can act as referral units for nephrologists from surrounding primary and secondary healthcare facilities. The suggested items included in the referral for a kidney–bone MDT are provided in Supplementary data. Several healthcare providers have pioneered the integration of MDTs into CKD-MBD management, and the collective experience has resulted in the identification of some general principles that can guarantee success (Fig. 5).

The most common indication for a bone biopsy in CKD-MBD is the determination of bone turnover. However, recent studies demonstrate that bone turnover markers often can differentiate high from low turnover with sufficient precision for clinical decision making [36, 37]. Additionally, they have the advantage of identifying dynamic changes of bone turnover in response to therapy. While the TMV classification includes the evaluation of bone volume, determination of BMD by DXA or by quantitative computed tomography are better validated for clinical fracture risk management. Thus, in many patients a treatment strategy may be decided without a bone biopsy. However, at present, identifying a mineralization defect still requires a bone biopsy. Developing local expertise to perform biopsies in reference centers with CKD-MBD MDTs, the application of small needle biopsies, and bone histomorphometry performed in reference laboratories may make it more feasible to obtain bone biopsies, when needed.

In conclusion, the present special report demonstrates how a kidney–bone MDT with expertise in CKD-MBD and osteoporosis may improve and expand the approach to diagnosis and treatment of CKD-associated osteoporosis, thereby improving fracture risk management and contributing to closing the treatment gap in patients with CKD.

## Supplementary Material

gfae197_Supplemental_File

## Data Availability

The data underlying this article cannot be shared publicly due to the privacy of individuals that are described in the cases. Approved consent from the individual cases has been achieved. If consent could not be achieved, the case has been modified to anonymize the case without any influence on the educational message of the case.
