# Supplementary material for: Multidisciplinary team approach for CKD-associated osteoporosis
Source: Nephrol Dial Transplant. 2024 Sep 24;40(1):48–59. doi: 10.1093/ndt/gfae197 (PMC11852330; doi:10.1093/ndt/gfae197)
Supplement: gfae197_Supplemental_File [file gfae197_Supplemental_File.docx]

**Supplementary material**

*The multidisciplinary team approach, a novel standard for the management of patients with CKD-associated osteoporosis: A special report by the European Renal Osteodystrophy (EUROD) initiative of the ERA CKD-MBD Working Group*

**Items to be included in a referral to the kidney-bone MDT**

Age, sex, menopause

**Relevant history**

Cause and stage of CKD.

Transplantation: prior transplantation(s) and use of corticosteroids, waitlisting

Dialysis vintage and modality, including any dialysis treatment prior to transplantation. Calcium bath concentration

Dynamic of current and previous disturbances of mineral metabolism (hyper-/hypoparathyroidism, mineralization defects, previous bone biopsy results, etc.)

Comorbidity (especially diseases affecting bone: Diabetes? Rheumatology? Cardiovascular disease? Osteoporosis? Malignancies? Malabsorption? Etc.)

Family history of fractures

Previous fractures and falls

Cardiovascular disease (Ischemic heart disease and myocardial infarction, peripheral artery disease and amputation, stroke)

Calciphylaxis

Previous parathyroidectomy (adenoma, hyperplasia)

Special diet (affecting calcium and/or vitamin D intake i.e., vegan diet etc.)

Height, previous height

Results of bone biopsies, if present

Dental health

**Medications** (previous and current)

Corticosteroids (cumulative dose, frequency and duration of periods with high doses)

Anti-osteoporotic medication (response to these and reason for withdrawal)

CKD-MBD: Active and nutritional vitamin D, calcimimetics, phosphate binders, calcium (response to these and reason for withdrawal)

Any other medications affecting the skeleton (e.g. diuretics, PPI, immunosuppressive or antineoplastic treatment, etc.)

**Biochemisty** (relevant dynamic changes must be included)

CKD-MBD: PTH, calcium, phosphate, 25OH vitamin D, magnesium, alkaline phosphatase (any other non-routine biochemistry, e.g. calcitriol, sclerostin, and FGF-23)

Bicarbonate, acidosis?

Bone turnover markers if available: bone specific alkaline phosphatase, iP1NP, TRAcP-5b, CTX

M-component, immunoglobulines, light chains

TSH

Female: estradiol, FSH, LH

Male: testosterone, FSH, LH, PSA

**Imaging**

X-ray of thoraco-lumbar spine

Fracture analysis of CT of thorax and/or abdomen

Dual Energy X-ray (DXA): T-score and Z-score and their changes

If present: pQCT and HR-pQCT

**Other**

Any other information relevant for the case, e.g. low birth weight, exposure to environmental or occupational hazards affecting the skeleton, etc.
